# Effect of light intensities on the photosynthesis, growth and physiological performances of two maple species

**DOI:** 10.3389/fpls.2022.999026

**Published:** 2022-10-12

**Authors:** Jinfeng Zhang, Jingru Ge, Buddhi Dayananda, Junqing Li

**Affiliations:** ^1^ Beijing Key Laboratory for Forest Resources and Ecosystem Processes, Beijing Forestry University, Beijing, China; ^2^ Optoelectronic College, Beijing Institute of Technology, Beijing, China; ^3^ School of Agriculture and Food Sciences, The University of Queensland, Brisbane, QLD, Australia

**Keywords:** maple, light intensity, photosynthetic, morphological, physiological

## Abstract

Photoinhibition decreases photosynthetic capacity and can therefore affect the plant survival, growth, and distribution, but little is known about how it affects on kindred tree species. We conducted field experiments to measure the photosynthetic, growth and physiological performances of two maple species (*Acer mono* and *A. pseudosieboldianum*) seedlings at four light intensities (100%, 75%, 55%, and 20% of full light) and evaluated the adaptability of seedlings. We found that: (1) *A. mono* seedlings have larger light saturated photosynthetic rates (*A*_max_), the light saturation point (LSP), and lower light compensation point (LCP) than *A. pseudosieboldianum* seedlings, thus indicating that the former has a stronger light utilization ability. (2) *A. mono* seedlings under 75% light intensity and had higher seedling height (SH), basal stem diameter (BSD), leaf number (LN), leaf area per plant (LAPP) and total dry weight (TDW), while *A. pseudosieboldianum* seedling at 55% light intensity displayed greater growth advantages, which agreed with their response of light saturated photosynthetic rate. Morphological plasticity adjustments such as decreased root shoot ratio (RSR) and increased specific leaf area (SLA) showed how seedlings adapt to weak light environments. (3) 100% and 20% light intensities increased the malondialdehyde (MDA) content of two maple seedlings, indicating that very strong or very weak light could lead to the imbalance of reactive oxygen species (ROS) metabolism. The regulation of antioxidant enzyme activities such as superoxide dismutase (SOD), peroxidase (POD) and catalase (CAT), as well as the content of osmoregulation substances such as free proline and soluble protein, are the main mechanisms of plant adaptation to light stress. Although both *A. mono* and *A. pseudosieboldianum* are highly shade tolerant, subtle differences in the photosynthetic, morphological and physiological traits underpinning their shade tolerance suggest *A. pseudosieboldianum* has the advantage to deal with the light threat. Future studies should focus on the expression level of photosynthesis-related genes and cell, to better understand the adaptation mechanism of plants to light variation which facilitates forest development, either natural or *via* silvicultural practices. This information expands our understanding of the light-regulating mechanism of trees, which contributes to develop management practices to support natural forest regeneration.

## Introduction

Photoinhibition often occurs when light energy is excessive, which reduces photochemical efficiency and even causes photooxidative system damage (Ma et al., 2015; Dias et al., 2018). Furthermore, low light intensity influences photosynthesis, which is central to plant productivity, and can therefore severely restrict plant growth (Zhu et al., 2014), and even death (Wang et al., 2021). During the evolutionary process, plants had various adaptive strategies to decrease the potential damage caused by light stress (Walters et al., 1993). Many studies have shown that plants can reduce the direct absorption of light energy by modifying morphological and photosynthetic plasticity, such as decreasing specific leaf weight (SLW), increasing specific leaf area (SLA) or enhancing light utilization capacity through the reduction in the light saturation point (LSP) and lower light compensation point (LCP) (Kaelke et al., 2001; Zhu et al., 2014; Sugiura et al., 2016). Moreover, plant species can adjust their physiological characteristics in response to the variation in light intensity. For example, high levels of antioxidant enzyme activity which enable the rapid clearance of reactive oxygen species (ROS) (Ma et al., 2015; Ozturk et al., 2021). Similarly, osmoregulation substances also play a key role in protecting plants from injury (Kishor et al., 2005; Kučerová et al., 2019).

The early growth and survival of seedlings are very important for their successful supplement into the young tree stage, and light intensity plays a determinant role in this stage (Loik and Holl, 1999; Razzak et al., 2017). However, in forest development and succession, the light environment varies greatly at both temporal and spatial scales (Avalos and Mulkey, 2014). For example, the destruction and fragmentation of forests are bound to cause sharp changes in light intensity, which may not be beneficial for the regeneration of many trees (Paquette et al., 2012; Yao et al., 2014). Even in the forest, the distribution of light is uneven due to the gap and stratification (Popma and Bongers, 1988; Tripathi et al., 2020). The adaptability of seedlings to different light environments may determine the status of the tree species in the forest community (Valladares et al., 2002; Rabara et al., 2017). In addition, previous studies on seedlings in canopy gaps or forest edges suggest that native tree seedlings may be inhibited by high light (Yu and Hao, 1998; Wu et al., 2006).

Maple trees, *Acer mono* and *Acer pseudosieboldianum*, belong to the Aceraceae family, which are late succession and shade-tolerant species widely distributed in the natural mixed-broadleaved Korean pine forests in Changbai Mountains, Northeast, China (Ye et al., 2014). These two maple trees are also widely used in landscape architecture construction due to their bright colors (Xie et al., 2021). Previous field investigations found that numerous *A. mono* has developed into the dominant species in the main story, while *A. pseudosieboldianum* is the most important constructor in a forest sub-story (Zhu et al., 2007; Ye et al., 2014; Zhang et al., 2015). Both maple trees are shade tolerant and kindred species, but they have different distribution patterns and abundances in the forest, which may be caused the differentiation in light requirements for the establishment and growth of seedlings (Paquette et al., 2012). Hence, the identification of light requirements is necessary to understanding the regeneration of tree species and facilitating forest development, either natural or *via* silvicultural practices.

Here, we investigated the light acclimation capacity of *A. mono* and *A. pseudosieboldianum* seedlings in response to light conditions, and we hypothesized that: 1) *A. mono* seedlings may exhibit high photosynthetic efficiency under high light, while *A. pseudosieboldianum* seedlings may be limited. 2) The photosynthetic, morphological and physiological traits underpinning seedlings’ shade tolerance may give *A. pseudosieboldianum* an advantage in coping with light threats.

## Materials and methods

### Seed collection and seedling propagation

We collected, *A. mono* and *A. pseudosieboldianum* seeds from mixed-broadleaved Korean pine forests (127°40’~128°16’ E, 41°35’~42°25’ N) in Changbai Mountains Northeast, China, from late September to early October 2020. Twenty independent individual maple trees were selected. The wings of the seeds were removed during seed collection, and the seeds were soaked in warm water at 45°C (initial temperature) in the laboratory to break the dormancy. The soaked time lasts for 7 days, and the water was renewed every 12 hours. The seeds were mixed with the appropriate amount of sand and put into a pot (30 cm inner diameter, 35 cm height, with good air permeability). Then, the pot with seeds was buried in the ground at 60 cm depth.

We dug out the pots with seeds on April 10, 2021, and then separated the seeds from the pots. The seeds were soaked in 0.5% KMnO_4_ solution to disinfect for 3 h, and sterilized seeds were thoroughly rinsed with purified water. The seedbed was built at the Northeast Asia botanical garden in Changbai Mountains. For the seedbed soil disinfection, 1:1500 phoxim was used for insecticidal treatment, then 1:500 carbendazim was used for sterilization, and sowed seeds on 15 April.

### Experimental design

To obtain light transmittance, photosynthetically active radiation (PAR) sensors (S-LIA-M003) with HOBO Micro Station Loggers (H21-002) (Onset Computer Corporation, USA) were installed in the forest gap, forest edge and understory of mixed-broadleaved Korean pine forests. The time step for data recording was set at 30 minutes. The light transmittance was calculated according to the following formula:


$$\begin{array}{l} \text{Light transmittance=}\\ \frac{\text{Photosynthetically active radiation under shade}}{\text{Photosynthetically active radiation under full light}}\times 100\% \end{array}$$

Four light intensity gradients were set up with different layers of black shade nets in the Northeast Asia botanical garden of Changbai Mountains, Northeast, China. The setup with 100% full light (L_100_) served as a control, three weak light intensities were set up according to the light transmittance to simulate the forest gap, forest edge and understory. Three weak light intensity treatments were 75% (L_75_), 55% (L_55_), and 20% (L_20_) of full light, which were set up with one layer, two layers, and three layers of nets, and each layer of shading net had three holes. In addition, branches from neighboring trees overtopping the experimental area were removed to secure homogeneous illumination.

On June 5, 2021, the healthy and homogenous seedlings (mean height of *A. mono* and *A. pseudosieboldianum* were 19.42 ± 5.32 cm and 21.32 ± 7.57 cm respectively, mean ± SD) were transferred to twenty plastic pots (20 cm inner diameter, 25 cm height, with holes in the bottom, six seedlings per pot) filled with a mixture of black soil, sand, branny, and pearlite (2:2:1:1, v/v/v, 40 kg m^-3^). During the first 15 days, all pots were placed in the built layers shading net for seedling retarding. On July 20, 2021, twenty plastic pots were randomly divided into four groups with five repetitions in each group and moved into the shade nets. In the early stages of the trial, the seedlings were watered every two days.

### Photosynthetic measurements

Fully developed leaves (the second, third and the fourth from the top) of three robust seedlings of each tree species were randomly selected under each light environment from August 18 to 25, 2021. Photosynthesis (*P*_n_) was measured using a portable photosynthesis system (LI-6400, LiCor, Lincoln, NE, USA) at 10 levels of the photosynthetic photon flux density (PPFD), starting from 0, then 40, 60, 80, 100, 150, 200, 400, 600, 800, and 1200 μmol·m^-2^·s^-1^. During the measurements, the ambient CO_2_ concentrations, the temperature of the leaf chamber and air relative humidity were fixed for 380 μmol·mol^–1^, 30°C and 50% respectively. The data were recorded between 8:30 and 11:30 a.m. To fit the photosynthetic light-response curve, we used the non-rectangular hyperbolic photosynthetic model proposed by Ye et al. (2013). The light-saturated photosynthetic rates (*A*_max_), LCP, LSP and dark respiration rate (*R*_d_) were derived from the photosynthetic light-response curve.

### Morphological measurements

We harvested seedlings on October 15, 2021. Each seedling, together with its taproots, was carefully removed from the soil, placed into sealed bags, and then transported to the laboratory. The seedlings were carefully washed with tap water and dried using filter papers. The number of leaves in the seedlings was counted, and the seedling height (SH) and basal stem diameter (BSD) were measured using a vernier caliper. The leaf area per plant (LAPP) was measured by a scanner (Canon scan lide120) and analysed using image analyzer (Image J). The seedlings were sorted into leaves, stems, and roots and subsequently dried in a dry oven at 85°C for 48h until constant mass, and then weighed with an electronic balance (EX224ZH 1/10000g; Ohaus Instruments, Changzhou, China). The total dry weight (TDW), root shoot ratio (RSR) and SLA were calculated based on Kelly et al. (2015):


$$\begin{array}{c} \text{Total dry weight (TDW)=}\\ \text{Root dry weight+Stem dry weight+Leaf dry weight (g)} \end{array}$$

$$\text{Root shoot ratio (RSR) = }\frac{\text{Root dry weight}}{\text{Stem dry weight+leaf dry weight}}$$

$$\text{Specific leaf area (SLA) = }\frac{\text{Leaf area}}{\text{Leaf dry weight}}({\text{cm}}^{2}\text{/g)}$$

### Physiological measurements

Leaves of two maple seedlings were randomly selected from one pot per treatment on September 1, 2021. The leaves were cut and mixed, they were randomly divided into three groups as three repetitions. The activities of superoxide dismutase (SOD), peroxidase (POD) and catalase (CAT) were determined by the guaiacol method (Beauchamp and Fridovich, 1971), UV absorption method (Thomas et al., 1982), and azoblue tetrazole photoreduction method (Díaz-Vivancos et al., 2008). The content of malondialdehyde (MDA), soluble protein and free proline were determined by the thiobarbituric acid technique (Deng et al., 2012), Coomassie Brilliant Blue G-250 method and ninhydrin staining (Bates et al., 1973; Kučerová et al., 2019).

### Data analysis

We used the One-way ANOVA to analyze the differences in photosynthetic, morphological and physiological parameters of the two species under different light intensities and the differences between different species under the same light intensity, and Duncan’s multiple range test was used to detect differences between means. All analyses were performed within SPSS (Version 21.0) and Origin 2019.

## Results

### Light response curves

**Figure 1:**
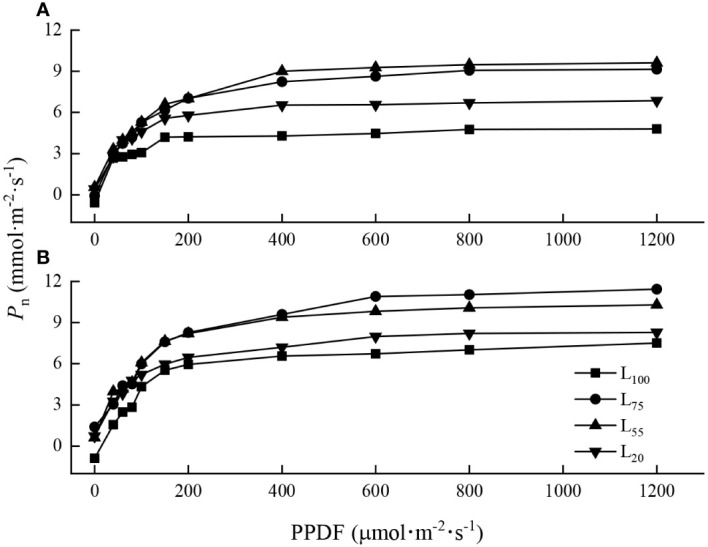
Light-photosynthetic response curves of two maple seedlings under different light intensities. **(A)**, *A. pseudosieboldianum*; **(B)**, *A. mono*.

The light response curves of maple seedlings varied with species. When PPDF< 200 μmol·m^-2^·s^-1^, the light response curves of the two species under different light intensities were similar, and the *P*_n_ increased sharply with the increase of PPDF (**Figures 1A, B**). When PPDF>400 μmol·m^-2^·s^-1^, the *P*_n_ of *A. pseudosieboldianum* seedlings tended to be stable and reached the LSP (**Figure 1A**). The *P*_n_ of *A. mono* tended to be stable when the PPDF>600 μmol·m^-2^·s^-1^ (**Figure 1B**).

### Photosynthetic parameters

The two maple seedlings exposed to 100% intensity showed the lowest *A*_max_ (**Table 1**). The *A*_max_ of *A. pseudosieboldianum* seedlings was the highest under 55% intensity, while that of *A. mono* was the highest under 75% intensity. With the decrease in light intensity, the LSP of *A. pseudosieboldianum* seedlings decreased gradually, and the LSP under 100% intensity was significantly greater than 75%, 55% and 20% intensity (*P<* 0.05); A similar response was observed for *A. mono* seedlings, but it was not significant under the different light intensities. Compared with the 100% light intensity, 75%, 55% and 20% intensity decreased LCP for two species, and the LCP of *A. mono* seedlings under 100% intensity was significantly greater than 75%, 55% and 20% intensity (*P<* 0.05). The *R*_d_ of two maple seedlings decreased gradually with the increase of light intensity, but a significant difference was not observed.

**Table 1:** Photosynthetic characteristics of two maple seedlings under different light intensity treatments.

Species	Treatments	*A*_max_(µmol•m^-2^•s^-1^)	LSP(µmol•m^-2^•s^-1^)	LCP(µmol•m^-2^•s^-1^)	*R*_d_(µmol•m^-2^•s^-1^)
*A. pseudosieboldianum*	L_100_	4.50 ± 0.85^b^	2.84 ± 0.88^a^	16.57 ± 6.20^a^	1.08 ± 0.46^a^
	L_75_	8.86 ± 2.12^a^	1.36 ± 0.46^b^	14.25 ± 4.44^a^	0.98 ± 0.20^a^
	L_55_	9.47 ± 1.89^a^	1.17 ± 0.48^b^	14.13 ± 3.26^a^	0.91 ± 0.13^a^
	L_20_	6.63 ± 2.69^ab^	1.12 ± 0.37^b^	12.91 ± 4.85^a^	0.92 ± 0.32^a^
*A. mono*	L_100_	7.05 ± 1.85^b^	2.96 ± 0.65^a^	18.17 ± 7.55^a^	1.14 ± 0.42^a^
	L_75_	11.143 ± 3.96^a^	2.47 ± 0.51^a^	13.86 ± 4.09^b^	0.89 ± 0.28^a^
	L_55_	10.03 ± 3.52^a^	2.38 ± 0.75^a^	11.70 ± 3.45^b^	0.92 ± 0.34^a^
	L_20_	7.97 ± 2.88^b^	2.28 ± 0.60^a^	13.25 ± 5.25^b^	0.88 ± 0.21^a^

A_max_, the light-saturated photosynthetic rate; LSP, light saturation point; LCP, light compensation point; R_d_, dark respiration rate. Small letters indicate significant differences under different light intensities (P< 0.05).

### Morphological characters

The shading was beneficial to the growth of two maple seedlings. For example, 55% light intensity resulted in the highest SH, BSD, LN, LAPP, and TDW of *A. pseudosieboldianum* seedlings, and the seedlings under 75%, 55%, and 20% light intensity were significantly higher than those under 100% light intensity (*P<* 0.05) (**Table 2**). The SH, BSD, LN, and TDW of *A. mono* seedlings under 75% and 55% light intensity were significantly higher than those under 100% and 55% light intensity (*P<* 0.05), and the LAPP was significantly different under different light intensity (*P<* 0.05). Two maple seedlings showed decreased RSR in response to dropped light intensity while the SLA increased (**Table 2**).

**Table 2:** The growth parameters of two maple seedlings under different light intensity treatments (mean ± SD).

Species	Treatments	SH	BSD	LN	LAPP	TDW	SLA	RSR
*Acer* *pseudosieboldianum*	L_100_	293.99 ± 20.86^b^	2.65 ± 0.40^a^	6.75 ± 0.38^a^	31.29 ± 2.32^b^	0.59 ± 0.05^a^	346.48 ± 25.29^b^	1.52 ± 0.10^a^
	L_75_	350.41 ± 9.92^a^	2.85 ± 0.74^a^	7.53 ± 0.23^a^	44.12 ± 5.35^a^	0.75 ± 0.12^a^	384.29 ± 40.37^b^	1.39 ± 0.15^ab^
	L_55_	369.57 ± 31.46^a^	3.28 ± 0.57^a^	7.65 ± 0.77^a^	51.44 ± 6.50^a^	0.86 ± 0.21^a^	389.38 ± 45.49^b^	1.22 ± 0.24^b^
	L_20_	344.65 ± 20.82^a^	3.15 ± 0.26^a^	7.85 ± 1.57^a^	44.32 ± 12.33^a^	0.68 ± 0.09^a^	453.48 ± 48.29^a^	1.00 ± 0.17^c^
*Acer mono*	L_100_	344.33 ± 24.36^c^	2.70 ± 0.25^b^	6.00 ± 0.33^c^	41.22 ± 4.22^d^	0.61 ± 0.09^b^	268.83 ± 45.01^b^	1.29 ± 0.33^a^
	L_75_	408.21 ± 35.23^a^	3.02 ± 0.12^a^	10.10 ± 0.99^a^	66.57 ± 2.09^a^	1.03 ± 0.13^a^	287.90 ± 50.47^ab^	1.09 ± 0.31^a^
	L_55_	400.29 ± 12.07^a^	3.23 ± 0.18^a^	9.25 ± 0.50^a^	58.51 ± 2.83^b^	0.88 ± 0.22^ab^	309.59 ± 37.77^ab^	0.96 ± 0.32^a^
	L_20_	372.89 ± 13.47^b^	2.85 ± 0.19^b^	7.30 ± 0.38^b^	49.02 ± 3.71^c^	0.67 ± 0.12^b^	349.47 ± 29.82^a^	0.92 ± 0.31^a^

SH, seedling height; BSD, basal stem diameter; LN, leaf number; LAPP, leaf area per plant; TDW, total dry weight; RAS, root shoot ratio; SLA, specific leaf area. Small letters indicate significant differences under different light intensities (P< 0.05).

### Antioxidant enzymes activity and MDA content

**Figure 2:**
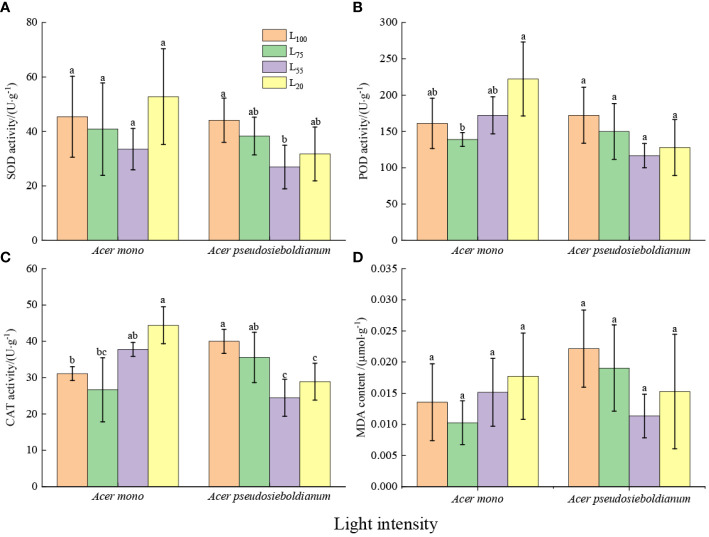
Effect of light intensity on leaf antioxidant enzymes (**A**, SOD; **B**, POD; **C**, CAT) and MDA **(D)**. Small letters indicate significant differences under different light intensities (*P* < 0.05).

The SOD activity of *A. mono* seedling seedlings under 20% and 100% light intensity was higher than that under 55 and 20% light intensity(significance was not observed); and the SOD activity of *A. pseudosieboldianum* seedlings under 100% light intensity was significantly higher than 55% light intensity (*P<* 0.05) (**Figure 2A**). Compared with the 100% light intensity, 75% light intensity decreased POD and CAT activity, while 55% and 20% light intensity increased POD and CAT activity of *A. mono* seedlings, especially 20% light was significantly higher than 75% light (*P<* 0.05). Compared with 100% light intensity, 75%, 55% and 20% light intensity reduced the POD and CAT activity of *A. pseudosieboldianum* seedlings, and the CAT activity under 100% light was significantly higher than that of 55% and 20% light (*P<* 0.05) (**Figures 2B, C**). The 20% light intensity resulted in the lowest MDA content of *A. mono* seedlings, while the MDA content of *A. pseudosieboldianum* seedlings was the lowest when the light intensity was 100% (**Figure 2D**).

### Content of soluble protein and free proline contents

**Figure 3:**
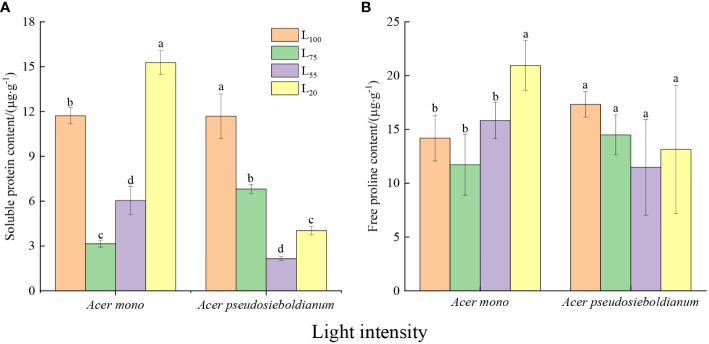
Effect of light intensity on osmoregulation substance (**A**, soluble protein; **B**, free proline contents). Small letters indicate significant differences under different light intensities (*P* < 0.05).

The soluble protein content of two maple seedlings was significantly different under different light intensities (**Figure 3A**, *P<* 0.05). Among them, 75% light intensity resulted in the lowest soluble protein content of *A. mono* seedlings, while the soluble protein content of *A. pseudosieboldianum* seedlings was the lowest when the light intensity was 55%. The free proline content of *A. mono* seedlings under 20% light intensity was significantly higher than 100%, 75% and 55%, but there was no significant difference among the latter three (**Figure 3B**).

## Discussion

### Photosynthesis

The light-photosynthetic response curve is the key to understand the photochemical efficiency and photochemical processes of plants (Loik and Holl, 1999; Razzak et al., 2017). We found that when PPDF > 400 μmol·m^-2^·s^-1^, *P*_n_ of *A. pseudosieboldianum* seedlings tended to be stable (**Figure 1A**) while *P*_n_ of *A. mono* seedlings tended to be stable when the PPDF>600 μmol·m^-2^·s^-1^ (**Figure 1B**). This result is consistent with our assumption that as PPDF availability increased, the *A. pseudosieboldianum* seedlings were difficult to absorb electrons through photochemical processes and on the contrary, *A. mono* seedlings could deal effectively with the increase in light energy. This variation modes of photosynthetic characteristics may be related to the inherent genetic physiological, and it is also the result of the long-term adaptation of tree species to the environment (Fariba et al., 2014). We also found that *A. mono* seedlings have higher *A*_max_, LSP and lower LCP than *A. pseudosieboldianum* seedlings in four light gradients (**Table 1**). This result suggests that the photosynthetic potential for *A. mono* is high, which may also be the reason why this tree species occupies the forest’s main storey in the natural mixed-broadleaved Korean pine forests. Furthermore, we found that the *A*_max_ of *A. pseudosieboldianum* seedlings was the largest at 55% light intensity, while *A. mono* seedlings exhibited the largest *A*_max_ at 75% light intensity (**Table 1**), reflecting that 55% and 75% of full light may be the optimum light levels for the two species respectively. In the field, the optimum light of *A. pseudosieboldianum* and *A. mono* is congruent with the habitat choice, which prefers forest gaps, forest edges, and the top of the canopy (Wu et al., 2006; Ye et al., 2014).

In this study, the *A*_max_ of two species under 100% light intensity was significantly lower than 75% and 55% treatments, indicating that the photosynthesis of maple seedlings was limited under strong light. This response to excess light energy is common in other shade tolerant species such as *A. Saccharum* (Marilou and Christian, 1998), *Pinus koraiensis* (Zhu et al., 2014), *Fagus grandifoli* (Collin et al., 2017), and *Quercus virginian* (Thyroff et al., 2019). Moreover, we found that the LSP and LCP of the two species dropped with the weakening of light intensity, which was consistent with the previous results showing the relatively low LCP and LSP of shade tolerant species were conducive to plants to utilize the light energy more efficiently under weak light environment, thereby increasing the accumulation of organic matter (Ma et al., 2015). Lower *R*_d_ is generally considered as the adaptive response of plants to cope with shaded conditions and obtain the maximum carbon benefit (Dias et al., 2018). *R*_d_ of two species under 75%, 55%, and 20% light intensity was lower than that of 100% treatment in our study, although not significant. This suggests that under shading conditions, seedlings reduce the loss of photosynthetic products and maintain the balance of carbon metabolism by decreasing *R*_d_, which was also confirmed by Yao et al. (2014) in the study of *Abies holophylla*.

### Seedling growth

Light is a key factor affecting the early growth of tree seedlings in the forest (Collin et al., 2017). Seedling regeneration may fail in shaded habitats with insufficient light (Dias et al., 2018). As a result, seedlings must rely on forest gaps or forest edges to achieve individual regeneration. Previous studies have shown that the greater the light intensity, the better the seedling growth (Gehring, 2003; Kelly et al., 2015), however, two maple seedlings exposed to 100% light intensity resulted in significantly lower SH and LAPP compared with the seedlings grown under the 75%, 55%, and 20% light treatments in this study, and BSD, LN and TDW also had a similar trend (**Table 2**). These results showed that full light has little benefit to the maple seedling growth and is expected that maple trees are reputed to be a late succession and shade tolerant species. Moreover, under the canopy of closed adult plants in the natural mixed-broadleaved Korean pine forests in Northeast China, maples often form a dense seedling bank with a state of growth inhibition, and these seedlings can survive for many years (Ye et al., 2014). Notably, SH, BSD, LN, LAPP, and TDW of *A. pseudosieboldianum* seedlings were the largest under 55% light intensity, while *A. mono* seedlings grew best under 75% light intensity (**Table 2**). The different growth responses of two species to the different light levels may be explained by the photosynthetic variables previously observed in our study, and thus, the optimum light intensity required for seedlings determines their growth. A similar result was also reported in *Camptotheca acuminata* (Ma et al., 2015) and *Tetracentron sinense* (Lu et al., 2020).

The modifying of morphological plasticity is an adaptive response of plants to environmental stress (e.g., drought, high salinity and shade) and is also an important way for plants to improve population fitness and resource acquisition ability (Kitajima, 1994; Tripathi et al., 2020). In the present study, we found that the SLA of *A. pseudosieboldianum* seedlings under 20% light intensity was significantly higher than that of 100% treatment, while the RSR under 20% light intensity was significantly lower than that of 100%, 75%, and 55% treatments (**Table 2**). Similarly, in *A. mono* seedling, the light of decreased intensity resulted in the increase of SLA and the decrease of RSR (**Table 2**). This morphological response to variation in light availability has been observed in many other studies (Popma and Bongers, 1988; Avalos and Mulkey, 2014; Tang et al., 2015). This may be the result of the trade-off between plant biomass aboveground and underground and light stress (Kitajima, 1994). Generally, soil moisture under strong light limits the upward extension of seedlings and eventually affects their growth and survival, thus seedlings allocate more photosynthetic products to the underground to form better developed roots, which is conducive to the absorption of water and nutrition; conversely, the biomass allocation of seedlings under weak light transferred to the aboveground, which can enhance the ability of plants to capture light (Walters et al., 1993; Kaelke et al., 2001; Kelly et al., 2015; Tang et al., 2015). Moreover, we found that SLA and RSR in *A. pseudosieboldianum* seedlings were higher than that of *A. mono* seedlings across the light intensity (**Table 2**). This result is consistent with Canham (1988) which found that shade tolerant species are generally more morphological plastic than less tolerant ones, which helps to improve the resistance and the ability to obtain resources of an individual tree seedling in the weak light environment, hence ensure the long-term reproduction of tree population (Paquette et al., 2012).

### Physiological characteristics

In stressful environmental conditions, the imbalance of ROS metabolism and the damage to the cell membrane system can lead to the increase of lipid peroxidation in biomembranes and permeability (Yi et al., 2020), thus resulting in the accumulation of MDA in leaf cells, the product of membrane lipid peroxide, and then decreasing the photosynthetic capacity (Ozturk et al., 2021). In this study, although a significant difference was not observed, the MDA content of two species under 100% and 20% light intensity was higher than that of 75% and 55% treatments (**Figure 2D**). This result agrees well with a recent study that shows full light and deep shade aggravate oxidative damage to lipid membranes (Wang et al., 2021). However, plants have a complete antioxidant enzyme system including SOD, POD, and CAT, which can avoid the damage caused by ROS (Tang et al., 2015). In this study, compared with 75% light intensity, 100% light intensity increased the activities of SOD, POD and CAT of two species (**Figures 2A–C**), indicating that the scavenging ability of ROS was enhanced in the full light environment. This result agrees with the report on olive trees by Sofo et al. (2004). Similar results were also observed under 20% light intensity and the 20% light intensity enhances antioxidant enzyme activity of two species compared to 55% light intensity (**Figures 2A–C**), which could be due to the fact that the seedlings suffer from light threat under 20% light intensity more grievous than that under 55% light treatment. As a result, seedlings are bound to improve the activity of antioxidant enzymes to resist light stress and reduce light damage (Ozturk et al., 2021).

Another immediate response of plants to cope with light stress is osmotic regulation (Ozturk et al., 2021; Wang et al., 2021). For example, free proline can stabilize the construction of membranes and protein by eliminating ROS (Bates et al., 1973; Kishor et al., 2005), and soluble protein protects cells against structural-metabolic disruptions and maintain osmolarity (Ozturk et al., 2021). In the present study, an obvious rise in the content of soluble protein and free proline of two species was observed under 100% and 20% light intensities (**Figures 3A and B**), which was consistent with the lower photosynthetic capacity under these two light intensities, indicating that the seedlings increase osmotic regulators to adapt full light and deep shade. Similar results were reported that high levels of soluble protein and free proline maintain cell stability and reduce high/low photo damage (Wang et al., 2021). It is worth noting that the proline and soluble protein content, as well as the above-mentioned three enzyme activities of *A. mono* seedlings, were the lowest at 75% light intensity, while these of *A. pseudosieboldianum* seedlings displayed a minimum at 55% light treatment (**Figures 2A–C** and **Figures 3A, B**), thus the subtle difference supporting their shade tolerance in the plasticity physiological shows that *A. pseudosieboldianum* more so than *A. mono*.

## Conclusion

Our work demonstrates that full light and deep shade limited the growth of two maple seedlings, the optimum light intensity for the growth of the *A. mono* and *A. pseudosieboldianum* seedlings was 75% and 55% of full light, respectively, which can account for the niche of two maple trees in the natural mixed-broadleaved Korean pine forests in Changbai Mountains, Northeast, China. On the other hand, the differentiation in light requirements improves a theoretical basis that in artificial seedling raising and management, appropriate shading should be given to ensure that they are in an optimal light environment. Moreover, while marked differences in growth exist in two maple species, the response in shade conditions is similar, such as increasing antioxidant enzyme activity or osmoregulation substance content, or increasing SLA and reducing RSR, and these responses guarantee the establishment of two tree species in long-term shaded environments. Future studies need to focus on the expression level of photosynthesis-related genes and cell structure, to better understand the adaptation mechanism of higher plants to light variation. Such information expands our understanding of the light-regulating mechanism of endangered plant species and contributes to develop management practices to promote natural forest regeneration.

## Data availability statement

The raw data supporting the conclusions of this article will be made available by the authors, without undue reservation.

## Author contributions

JL designed the research project and provided theoretical guidance. JZ collected and analyzed the data. JZ, JG, and BD wrote the manuscript. All authors contributed to the article and approved the submitted version.

## Funding

This research was funded by the National Science and Technology Basic Resources Survey Project (SQ2019FY101602).

## Acknowledgments

We would like to thank Shixiong Wu for his technical assistance.

## Conflict of interest

The authors declare that the research was conducted in the absence of any commercial or financial relationships that could be construed as a potential conflict of interest.

## Publisher’s note

All claims expressed in this article are solely those of the authors and do not necessarily represent those of their affiliated organizations, or those of the publisher, the editors and the reviewers. Any product that may be evaluated in this article, or claim that may be made by its manufacturer, is not guaranteed or endorsed by the publisher.
